# Drivers and Consequences of Size Declines in Unicells

**DOI:** 10.1111/ele.70387

**Published:** 2026-05-05

**Authors:** Dustin J. Marshall, Hayley E. Cameron, Akira Abe, Suzana Goncalves Leles, Craig R. White, John Delong

**Affiliations:** ^1^ School of Biological Sciences Monash University Melbourne Victoria Australia; ^2^ School of Biosciences University of Melbourne Melbourne Victoria Australia; ^3^ Marine Science Institute, University of Texas Austin Texas USA; ^4^ School of Biological Sciences University of Nebraska‐Lincoln Lincoln Nebraska USA

## Abstract

The communities of unicellular microbes (bacteria, protists and yeasts) that underpin ecosystems are changing. In warmer conditions, protists tend to shrink, but the consequences of these changes in size are unclear. We show preliminary evidence that warming‐mediated declines in cell size observed in protists also apply to bacteria and yeasts. Predicting the consequences of these warming‐mediated size declines requires that the relationships between cell size and key functional traits are well‐characterised. We show that the critical relationship between unicellular size and energy use—that is, metabolic scaling—has been systematically mis‐estimated in the past. Projections of the effects of warming on unicellular respiration change from superlinear to sublinear once the metabolic scaling relationship is updated, with worrying consequences for the biological carbon pump and other ecosystem services. Other size‐function scaling relationships (e.g., photosynthesis) are likely to have been similarly mis‐estimated. Next, we show that theory on the relationships between size, temperature and demography is more ambivalent than previously recognised, leaving uncertainty as to how warming will alter the dynamics of unicellular populations. Finally, we identify pathways for improving our capacity to predict future changes in unicellular size, and decrease the uncertainty surrounding the consequences of these changes.

## Graphical abstract

Previous syntheses concluded that metabolic allometric scaling is unusually steep (> 1) in unicells but here we show that this steepness is an artefact of laboratory studies that confound cell size with cell population density. Once this density effect is accounted for, metabolic scaling is ~0.86 in unicells. This finding changes predictions of how warming is predicted to affect fluxes in unicellular populations.

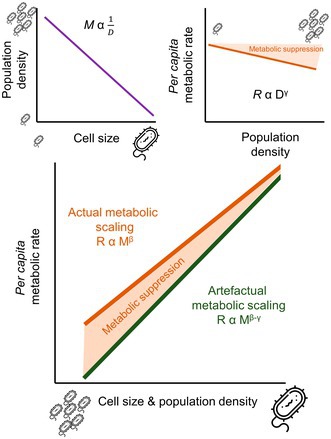

## 1 Introduction

Unicells (bacteria, protists and yeasts) are the most abundant life on the planet. Unicells underpin many of the biosphere's functions: driving diseases, biogeochemical cycles, and productivity (Czarnoleski and Verberk 2025). Thus, change in the abundance or functioning of unicells will have cascading effects on many biological processes and the ecosystem services upon which we rely (Ward and Follows 2016). The size of unicells is a key trait that drives their function and demography (Hatton et al. 2019; Czarnoleski and Verberk 2025): smaller cells need fewer resources (in absolute terms), so they tend to be more abundant than larger cells (Hatton et al. 2015). Unicells vary tremendously in size, across more than 9 orders of magnitude from the smallest to the largest, spanning a size range equivalent to that between an ant and an orca (Makarieva et al. 2008). This impressive variation across species has long fascinated biologists such that most syntheses of unicellular size focus on interspecific patterns (Makarieva et al. 2005; Marañón 2015). Cell‐size variation within species is equally important, but the drivers and consequences of this variation are far less studied.

**FIGURE 1:**
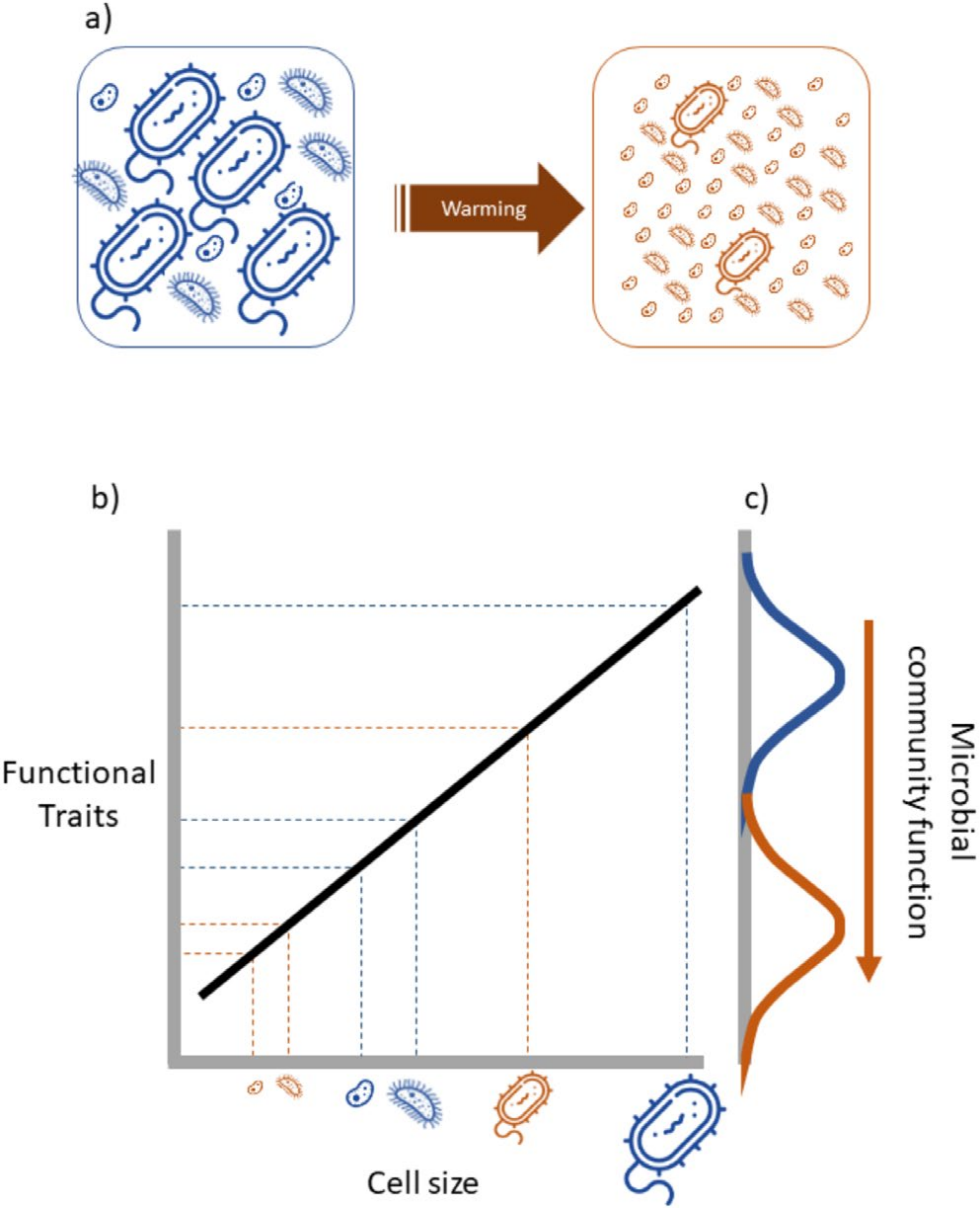
Schematic showing how average unicellular size may decrease, and functions could change with warming (shown in orange) relative to current day conditions (in blue). Panel (a) shows how average size can change in microbial communities via three (non‐mutually exclusive) processes: (i) shifts in the relative abundance of species of different sizes; (ii) cell size plasticity whereby species shrink; and (iii) selection for smaller cells within species. By estimating the relationships between cell size and functional traits both within and among species (panel b), we can then predict how the function of microbial communities will change with warming (panel c).

As climates warm, the sizes of unicells are changing: cells are getting smaller both within and among species. These size changes are manifesting across the diversity of unicellular life and across habitats, from soils (Sorensen et al. 2019) to lakes, rivers (Zohary et al. 2021) and oceans (Morán et al. 2015; Hillebrand et al. 2022). Thus, there is an urgent need to understand the extent and nature of changes in unicellular size, as well as their consequences. These consequences can only be predicted based on a good understanding of the relationships between cell size and function (Figure 1). Here, we review our understanding of unicellular size. First, we explore how warming changes the size of unicells and provide preliminary evidence for previously undiscovered patterns in bacteria and yeast. We then revisit canonical relationships between unicellular size and function, demonstrating that key relationships have been mis‐estimated, with implications for projections of the impacts of global change. Finally, we identify the theoretical and empirical pathways for meeting the challenge of predicting the trajectories of unicellular size changes and their consequences for population dynamics, biological fluxes and ecosystem functioning.

## 2 The Relationship Between Cell Size and Temperature

Warming can change the size distribution of unicellular communities at two scales of biological organisation simultaneously by: (i) altering the prevalence of species of different sizes; and (ii) altering the distribution of sizes within species (Figure 1). These processes are not mutually exclusive and may interact (Mousing et al. 2017), but for clarity, we will consider each scale separately, beginning with changes in cell size at the among‐species level.

While there are exceptions, there is a tendency for warmer temperatures to favour smaller‐celled species. For example, average cell sizes have decreased by ~1% each year since 2001 in Atlantic bacterial communities (Morán et al. 2015). Warming also increases the relative abundance of smaller species in freshwater systems (Zohary et al. 2021), marine phytoplankton (Hillebrand et al. 2022) and soil bacteria (Sorensen et al. 2019). This negative relationship between cell size and temperature has also been observed at larger spatio‐temporal scales: across both evolutionary time, and biogeographical regions (Mousing et al. 2017; Acevedo‐Trejos et al. 2018). While warmer communities generally favour smaller‐celled species, it is important to note that there are exceptions (for a nuanced discussion of this topic, see Hillebrand et al. 2022). Similarly, warming might not be the ultimate driver for cell size declines, instead, temperature could affect size indirectly via changes in resource abundance or fluxes (Deutsch et al. 2022; Hillebrand et al. 2022; Czarnoleski and Verberk 2025). Nevertheless, it seems reasonable to conclude that, for the most part, warming communities tends to increase the relative abundance of smaller‐celled species, such that mean cell sizes decrease with temperature.

Warming also decreases unicellular size within species via phenotypic plasticity. Some of the best evidence for temperature‐size relationships comes from protozoa and larger phytoplankton (see meta‐analyses by Atkinson et al. 2003; Forster et al. 2012; Hillebrand et al. 2022), which showed that cell volumes decrease by around 2.5% for every 1°C of warming. While most species seem to show a negative size‐temperature relationship, again we should note that not all relationships are negative nor linear: some species are known to increase in size when warmed, while others show a unimodal size relationship with temperature (Liang et al. 2019). Nevertheless, on average and within non‐stressful limits, cell sizes decrease with warming in many protozoa and phytoplankton species.

**FIGURE 2:**
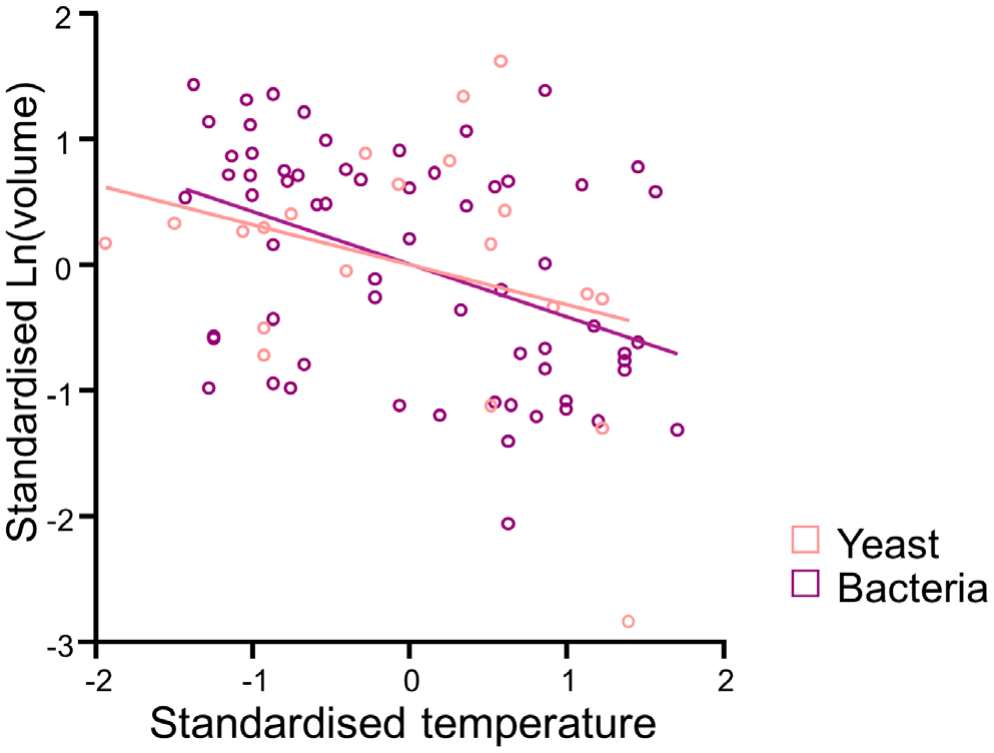
Higher temperatures decrease cell size within species of bacteria (blue) and yeasts (red). To present all data on a relative scale, both cell size and temperature are normalised within species ($\bar{x}=0$, SD = 1), each point represents a cell volume for a given temperature, and the line is the line of best fit for each group. See Supporting Information for within‐species reaction norms.

Notably absent from syntheses of intraspecific relationships between cell size and temperature are single‐celled fungi (i.e., yeasts) and bacteria. For example, the two largest syntheses exploring the effects of temperature on unicellular size each only include one bacterial species (Forster et al. 2012; Atkinson et al. 2003). More generally, cell size plasticity seems to be relatively less studied in bacteria, and compilations are lacking. To address this knowledge gap, we compiled and analysed data on how temperature affects cell size within species for yeasts and bacteria (see Supporting Information). We find that, while there are some exceptions (see Figure S1), bacteria and yeast tend to show a negative relationship between temperature and size on average (*F*_1,69_ = 4.62, *p* < 0.035). We found no evidence for differences in the relationship between cell size and temperature between yeasts and bacteria (Group × temperature interaction: *F*_1,68_ = 0.078, *p* = 0.781)—although admittedly we only had data for a few species of yeast, so more studies are needed for this group particularly. Within‐species, cells in warmer conditions are smaller than conspecifics in cooler conditions, decreasing in volume by ~1.5% for every 1 degree of warming (Temperature coefficient: −0.016 ± 0.006 SE; Figure 2). Thus, initial evidence suggests that bacteria and yeast show similar temperature‐size relationships to protists—warming tends to decrease cell size within species.

Warming alters the size of microbial communities both by increasing the relative abundance of smaller species and by decreasing cell size within each species. While few studies have quantified the relative contribution of both processes, some find that intraspecific changes in cell size account for around 70% of the drop in overall size (Mousing et al. 2017). Given these effects of warming on size at both the population‐ and community‐level, what are the consequences of size declines for the functioning and demography of unicellular populations and communities? To answer this, we need data on the relationship between size and function among species (for communities) and within species (for both populations and communities). Below we show that size‐function relationships at both scales remain unclear, albeit for different reasons. Interspecific relationships between unicell size and function have received the most attention, perhaps because such studies can leverage the remarkable range of sizes observed across species, and we will consider these first.

## 3 Cell Size‐Function Relationships Among Species and the Cautionary Tale of Metabolic Scaling

There are well‐documented relationships between unicellular size and resource uptake, photosynthesis and demography (Finkel 2001; Marañón 2015; Hatton et al. 2019; Hillebrand et al. 2022), but perhaps the best studied is that between unicellular size and metabolism (Makarieva et al. 2005, 2008; DeLong et al. 2010). Metabolic rate is a key trait because it sets the upper limit of many other functions—the maximum rate at which cells can acquire and use resources will be constrained by their metabolic rate. Consequently, metabolic rate also influences rates of population growth and maximum population density (Savage et al. 2004; Marshall et al. 2022). Thus, there has been perpetual interest in determining how unicellular size relates to metabolic rate, typically measured as oxygen consumption.

The scaling of metabolic rate, *R*, with mass, *M*, is described by the exponent *β*, such that:

(1) $$R\;\alpha \;M^{\beta }$$

In metazoans, the relationship between size and metabolic rate is sublinear, or hypoallometric (i.e., *β* ~0.75): larger species have higher absolute metabolic rates but relative to their mass, they have lower metabolic rates (Kleiber 1947). In other words, elephants consume more energy overall, but mice consume more energy per unit mass. In contrast, metabolic rate has been thought to increase more steeply with size in unicells. The relationship between size and metabolic rate has been argued to be linear in protists (*β* ~ 1 [Makarieva et al. 2008; DeLong et al. 2010; López‐Sandoval et al. 2014]); and superlinear in prokaryotes (*β* > 1 [Makarieva et al. 2008; DeLong et al. 2010; García et al. 2016])—that is, larger unicells not only have absolutely higher metabolic rates, they are also argued to have higher metabolic rates per unit mass. In other words, unicells are thought to have very different metabolic relationships to metazoans: mass‐specific metabolic rates are expected to *decrease* in metazoans, but *increase* in prokaryotes.

That unicellular life appears to have very different energetic scaling‐rules to multicellular life has sparked fierce debates about fundamental biological issues (reviewed in Muñoz‐Gómez 2024): from the energetics of genome complexity to the role of metabolic constraints in the evolution of eukaryotic life and hard limits on cell size (Kempes et al. 2012). Indeed, the finding of superlinear metabolic scaling in unicells was so unexpected, those who first documented it regarded their own result with skepticism (Makarieva et al. 2008). Subsequent analyses, however, appeared to confirm superlinear scaling relationships (DeLong et al. 2010; García et al. 2016): with some estimates being as high as 1.97, an exponent that almost quadruples metabolic rate with a doubling of cell size. Field studies also report superlinear metabolic scaling in prokaryote communities (Huete‐Ortega et al. 2012), with exponents ranging between 1 and 2 (García et al. 2016). Since these foundational compilations of unicellular metabolic scaling, more recent studies have come to a range of different conclusions (Table S2) such that the scaling of metabolic rate in unicells remains curiously unresolved.

Common to all of the above analyses is that they do not account for a potentially confounding effect when estimating the metabolic scaling of unicells: population density. Although cell size is an important driver of metabolism, it is not the sole driver: population density also influences per capita metabolic rate. Across a range of organisms and cell types, cells in higher population densities have lower per capita metabolic rates than conspecifics in lower densities (DeLong et al. 2014). This density‐dependence of metabolic rate may be a form of metabolic plasticity—cells may downregulate their metabolic demands at higher population densities because they are likely to experience more intense competition for resources. In this way, minimising metabolic rates at higher densities reduces per capita resource requirements, and may be a means through which populations can maximise their carrying capacity or persistence. These density effects can be strong: a recent meta‐analysis found that, on average, for every 10‐fold increase in cell density, per capita metabolism decreases by ~85% (Potter et al. 2024).

The effect of population density can modify estimates of metabolic scaling, obscuring the true relationship if left unaccounted for. Studies of microbial metabolic rate often use very different population densities (hereafter just referred to as ‘densities’) in their test samples, typically depending on the size of cells under investigation. Because smaller cells consume less oxygen per capita than larger cells, it is standard practice to load higher densities of smaller cells into respirometry chambers to detect a sufficient signal of oxygen consumption. Field studies share similar issues: smaller cells are much more abundant in nature than larger ones (Huete‐Ortega et al. 2012; Hatton et al. 2019; Atkinson et al. 2021). Hence, there is a strong, negative relationship between cell size and density in both the laboratory and the field when metabolic rates are measured (see also Figure S2).

Given that the metabolic rates of larger cells are measured at much lower densities than smaller cells, how might this alter estimates of metabolic scaling? Some simple theory provides explicit predictions. The per capita metabolic rate of cells (*R*_cell_) can be described by the power function:

(2) $$R_{\text{cell}}={\mathrm{aM}}^{\beta }$$

where *M* is cell mass, *β* is an exponent describing the scaling of mass with metabolic rate, and *a* is some species‐specific constant. Given that it can be difficult to measure the metabolic rate of individual cells, most studies estimate the metabolic rate of a sample of cells (*R*_sample_) as follows:

(3) $$R_{\text{sample}}={\mathrm{aM}}^{\beta }N$$

where *N* is the number of cells in the sample. Empirical estimates of *R*_sample_ are typically converted to *R*_cell_ by dividing by *N*, but this approach does not account for the effects of cell density on per capita metabolic rate. This can be done by incorporating a term for the effect of density, *D*, on metabolic rate according to the equation:

(4) $$R_{\text{cell}}={\mathrm{aM}}^{\beta }D^{\gamma }$$

where *γ* describes the scaling of metabolic rate with density (*γ* is typically negative [DeLong et al. 2014; Potter et al. 2024]). If we assume that empirical studies of microbial metabolism use densities that are inversely related to cell size (see Figure S2 for a confirmation of this assumption), such that *D α* 1/*M*, we can substitute Equation (4) into (3) to get:

(5) $$R_{\text{sample}}\;\alpha \;M^{\beta -\gamma }N$$

**FIGURE 3:**
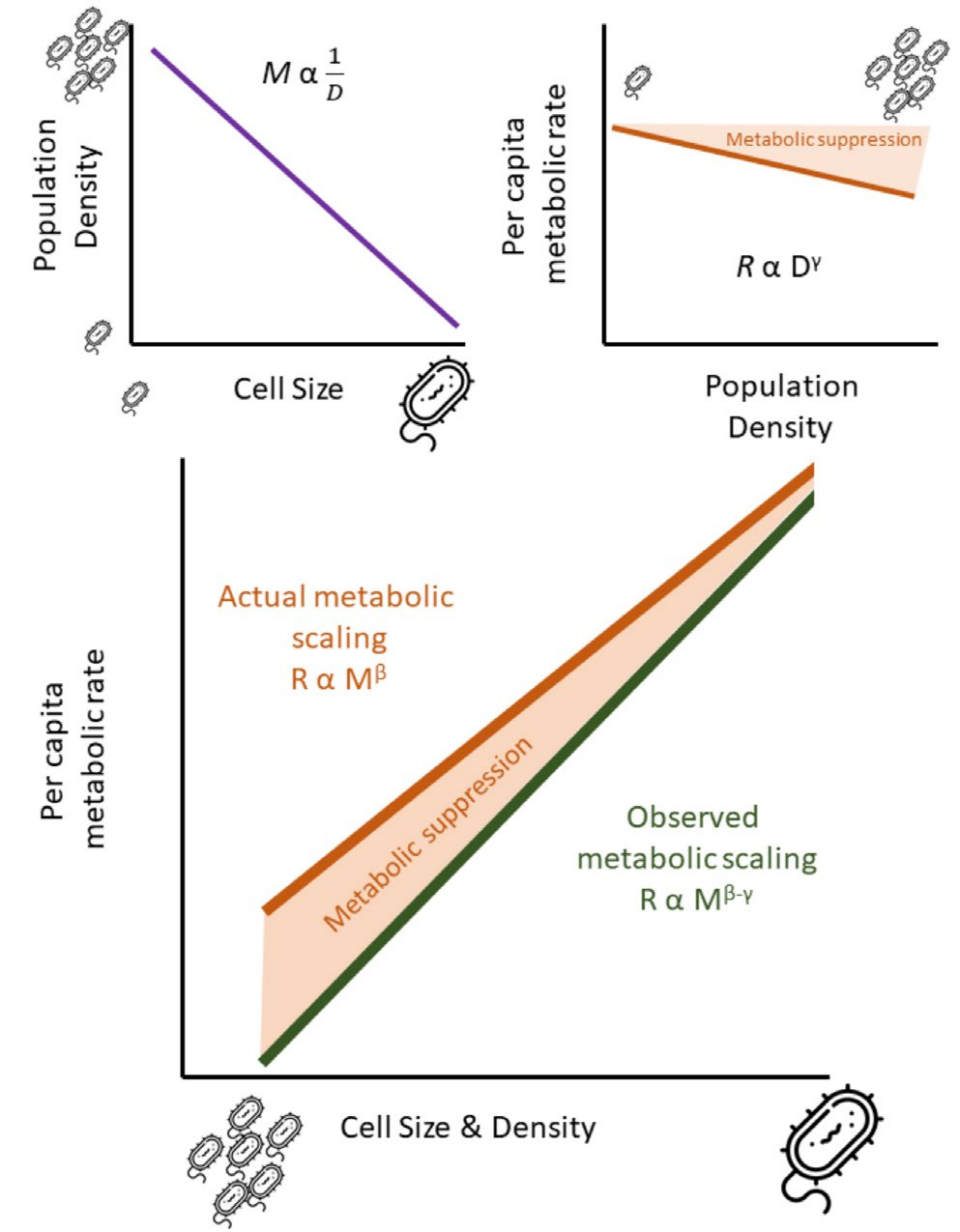
Schematic of the effect of density on estimates of metabolic scaling in unicells. Because there is likely to be a negative relationship between cell size (*M*) and population density (*D*; Top Left), and because higher densities suppress metabolic rates (top right), previous estimates of metabolic scaling that assume no effect of density may be overestimated (bottom).

Because traditional approaches use Equation (3) to estimate metabolic rates and fail to account for population density effects, then whenever γ ≠ 0, estimates of metabolic scaling will be mis‐estimated (see Supporting Information for a more general treatment where the relationship between *M* and *D* can take any form). Because γ is typically negative, estimates of metabolic scaling are likely to be systematically overestimated (Figure 3). Intraspecific compilations estimate *γ* ~ −0.8 (Potter et al. 2024), but interspecific estimates of γ (albeit across a smaller range of population densities) tend to be smaller (Malerba et al. 2017). Regardless, Equation (5) makes clear that if density has any effect on per capita metabolism, then it must be accounted for to generate unbiased estimates of metabolic scaling in unicells (Figure 3). Here, we update classic datasets (Makarieva et al. 2008) with more recent data, adding 34 new species including the dark respiration of unicellular autotrophs (see https://doi.org/10.5061/dryad.95x69p8xw for data), and then re‐analysed this augmented dataset (which included a total of 307 datapoints across ~240 spp.) to provide new estimates of metabolic scaling in unicells that explicitly account for the density‐dependence of metabolic scaling. For those species for which density was unreported, we then imputed density based on the relationship between cell size and density for those studies that did report density (see Supporting Information). Our estimates of metabolic scaling were the same whether we included these imputed densities or not. While previous syntheses tend to correct for temperature prior to analysing metabolic scaling relationships, here, we also included temperature as an additional predictor.

**FIGURE 4:**
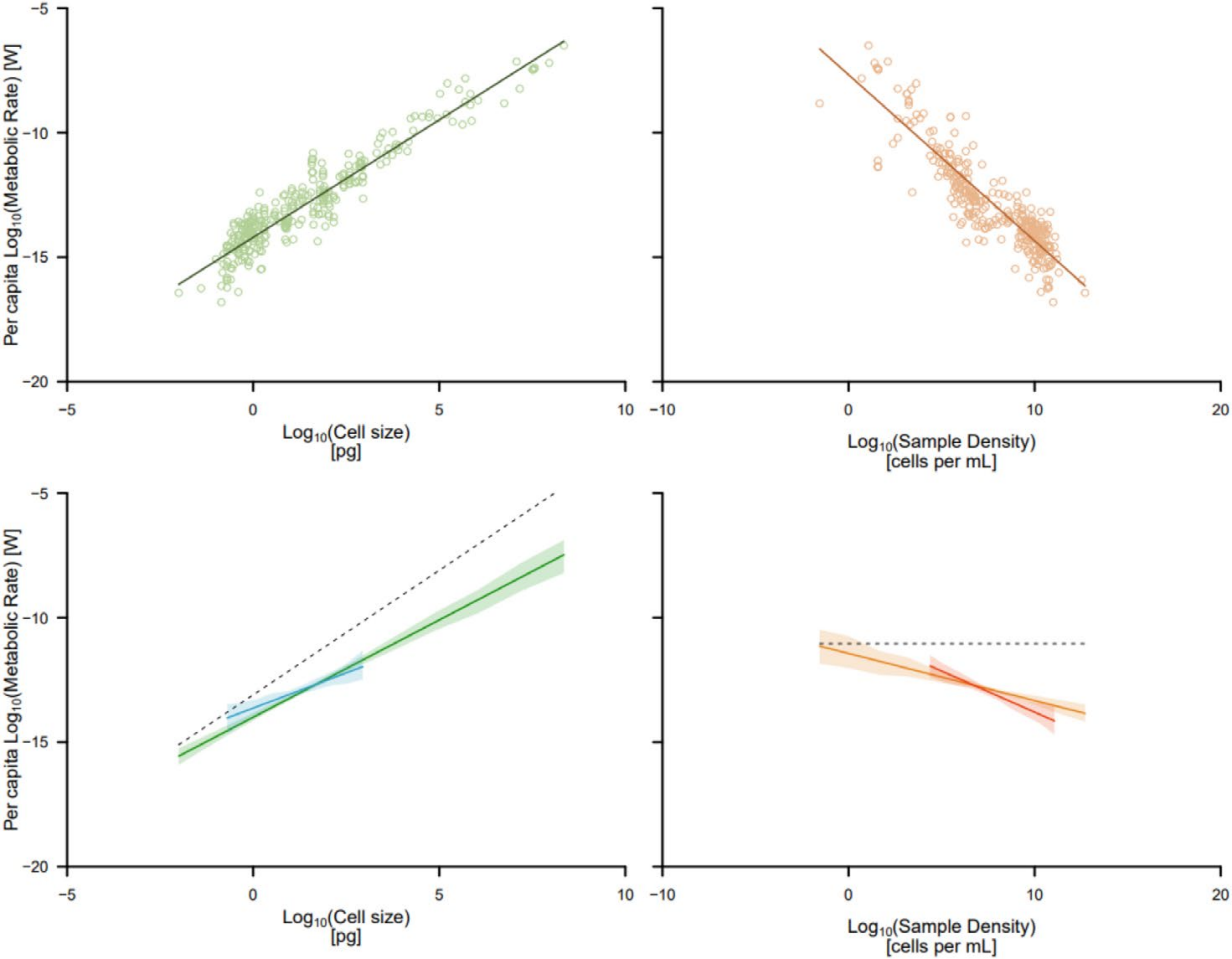
Relationships between per capita metabolic rate and cell size (left panels), per capita metabolic rate and sample density (right panels). The top panels show the raw relationships while the bottom panels show the true underlying relationships after statistically partitioning the relative contribution of each factor. In all panels the solid line is the line of best fit. In the bottom left panel, the dashed line shows a hypothetical metabolic scaling of 1, and in the bottom right panel, the dashed line shows the assumption of no density effect on metabolic rate. The shaded regions in the bottom panels show parametric bootstrap 95% confidence intervals (100,000 bootstraps). The shorter lines show the fits from the model that only included the 35 species for which metabolism was measured at multiple densities.

Across prokaryotes and eukaryotes, we find that once density effects are accounted for, unicellular metabolic rate scales with cell size sublinearly (*β* = 0.793, CI: 0.701, 0.885; Wald test difference from *β* = 1: *t*_303_ = 4.40, *p* < 0.001; Figure 4). This analysis overturns a core tenet of microbial energetics: that is, we find no evidence that metabolic scaling is much steeper in unicells relative to metazoans. We also found no evidence for frequently postulated differences in metabolic rate between prokaryotic and eukaryotic unicells (Groups × Log_10_[Cell Size]: *F*_1,301_ = 0.041, *p* = 0.839), or intercept (Groups: *F*_1,302_ = 0.363, *p* = 0.547; Figure 4): suggesting that bacteria are able to maintain equivalent metabolic scaling relationships to those of similarly sized eukaryotes, despite lacking membrane‐bound organelles (e.g., mitochondria). Re‐analysis of these data using a phylogenetic mixed modelling approach (Hadfield and Nakagawa 2010) implemented in the *brms* (Bürkner 2021) package of R, for the 108 species that could unambiguously be assigned to a dated phylogeny (Kumar et al. 2022) revealed a vanishingly small phylogenetic signal (phylogenetic heritability [equivalent to Pagel's *λ* (Hadfield and Nakagawa 2010)] was < 0.01), and yielded qualitatively and quantitatively similar results, so we present the non‐phylogenetic approach here as it contains the most species.

We found that imputing density for some species had no impact on our estimate of metabolic scaling—when we re‐analysed the data including only those studies that provided the densities at which metabolism was measured, our estimate of metabolic scaling was unchanged (*β* = 0.793; CI: 0.69, 0.897). In other words, our finding of sublinear metabolic scaling is not influenced by imputed values for cell density.

Per capita metabolic rate decreased at an average rate of ~30% for every 10× fold increase in density (*F*_1,303_ = 17.667, *p* < 0.001; *γ* = −0.161, CI: −0.236, −0.086; Figure 4). Metabolic rate increased with temperature (*F*_1,303_ = 11.695, *p* = 0.001), and had a Q_10_ of almost exactly 2 (i.e., a 2‐fold change for every 10°C increase in temperature). There was a tendency for density effects to be stronger at higher temperatures but the temperature × density interactions was marginally non‐significant (Temperature × Density: *F*_1,302_ = 3.848, *p* = 0.051). There was no evidence for a three‐way interaction between density, size and temperature (*F*_1,300_ = 0.22, *p* = 0.638), nor was there evidence for the interactions between size and temperature (*F*_1,301_ = 0.412, *p* = 0.521).

When we only included data for those species (*n* = 35) for which metabolic rates were measured at multiple densities (including species identity as a random effect to account for nonindependence), we again estimated metabolic scaling to be sublinear, but the exponent was even shallower (*β* = 0.591; CI: 0.322, 0.861), and density effects were even stronger (*γ* = −0.345, CI: −0.479, −0.21; Figure 4) than those estimated using the full dataset. While this within‐species analysis improves the precision of our estimate of metabolic scaling by reducing the collinearity between density and cell size, it does reduce its accuracy because it conflates within‐ and among‐species density effects on metabolic rate (see the next section for an in‐depth discussion of this issue). Thus, we favour the metabolic scaling estimates from the larger dataset but include estimates from the within‐species subset for completeness. Regardless of which analysis is used, our findings contradict the near‐canonical view that unicells show steeper metabolic scaling than metazoans.

Our finding that density alters estimates of metabolic scaling in unicells implies that many other size‐function relationships have been mis‐estimated in unicells. The scaling of function with cell size is likely to be overestimated whenever the following conditions are met: (i) cell function is density‐dependent; (ii) there is negative cell size‐density covariance across studies; and (iii) density is omitted from the statistical model. The first two conditions seem likely for a range of cell functions. For example, photosynthesis and resource uptake have been estimated to scale at around 0.87 and 1 respectively in phytoplankton (Marañón 2015; Hillebrand et al. 2022): but both show strong density‐dependence. Indeed, a preliminary study for 21 species of phytoplankton found that per capita photosynthetic rates decrease with density regardless of light level (i.e., γ for photosynthesis is also negative [Malerba et al. 2017]). Assays of photosynthesis and resource uptake may also require denser cultures of smaller cells to detect reasonable signals but this requires testing. We suspect that size‐scaling relationships of these (and other) critical rates in unicells have been overestimated in similar ways to those as we have demonstrated for metabolism here. It is thus premature to make conclusive statements about how unicell size scales with the most fundamental functions until future syntheses formally account for the potentially confounding effects of cell density.

Our recalibration of unicellular metabolic scaling has implications for how predicted changes in cell size will alter microbial energy dynamics and the global carbon flux in a warming world. Were we to use traditional (superlinear) estimates of metabolic scaling, we would predict that a decrease in unicell size should decrease mass‐specific metabolic rates. For example, under superlinear scaling, a 30% decline in bacterial cell size would yield a *decrease* in metabolism of ~30% for a population of smaller cells relative to a population of conspecifics of larger cells at an equivalent biomass. Under our revised estimate of sublinear metabolic scaling, a decrease in cell size of 30% will actually *increase* metabolic rates by 5%; including the direct effects of temperature on metabolism will only exacerbate these effects. Thus, respiration rates are likely to increase in ways that have not been anticipated by global carbon models (López‐Urrutia et al. 2006). Indeed, models of global carbon fluxes tend to overlook cell size reductions, and the concomitant changes to mass‐specific respiration (Ward and Follows 2016)—but these will have particularly worrying consequences for net carbon fixation, and hence the global carbon pump.

## 4 Relationships Between Cell Size and Function Within Species

**FIGURE 5:**
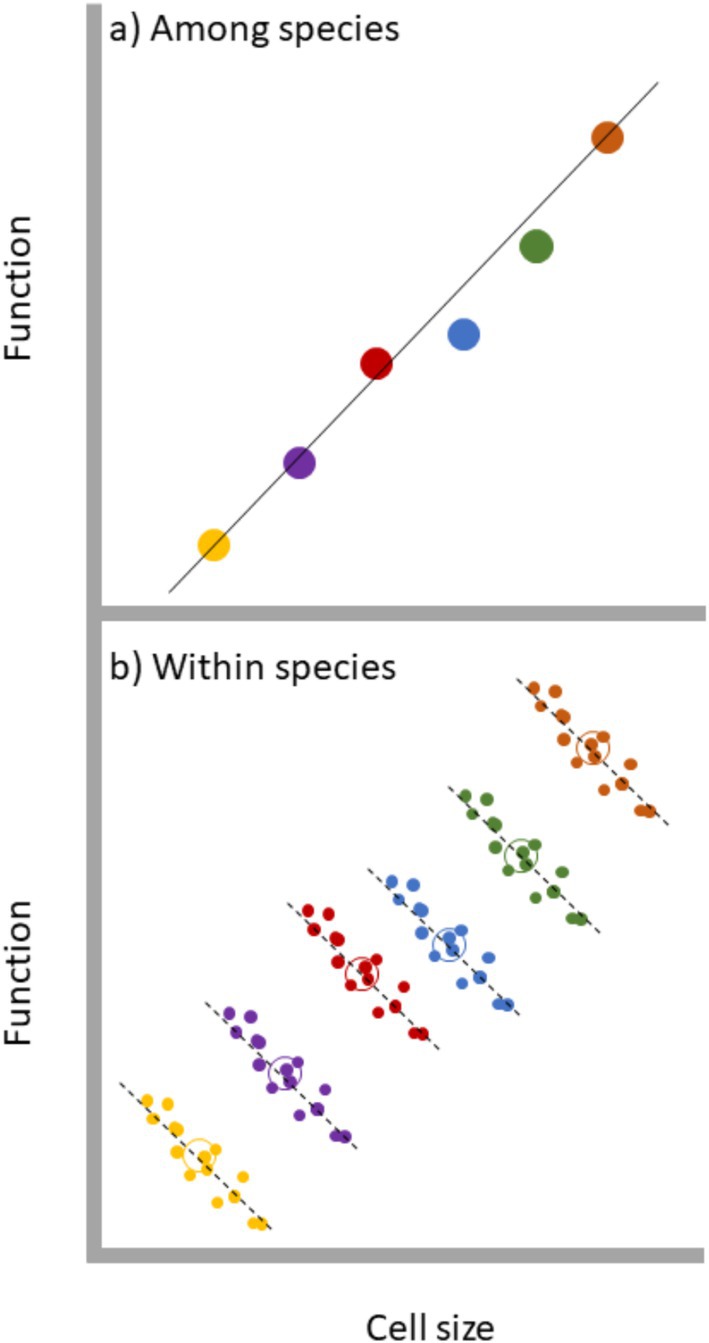
Schematic illustrating why among‐species covariances may not reflect within species covariances between cell size and cell function. In the hypothetical example, the relationship between cell size and function is positive among species but consistently negative within species. In panel (a) the circles depict the mean values of size and function for each species (shown by different colours) and the solid line represents the interspecific covariance. In panel (b) the within species data are depicted by filled circles for each species (shown again by different colours) and the dotted lines represent the intraspecific covariances. Note that the open circles show the centroids for each intraspecific covariance.

Our understanding of size‐function relationships within unicellular species is surprisingly limited for several reasons. First, it is important to recognise that we cannot use among‐species relationships as reliable proxies for within species relationships. While it is tempting to assume that the relationships between size and function among species recapitulate those same relationships within species (and some syntheses have conflated the two), there are good reasons to doubt this assumption. As counterintuitive as it may seem, an interspecific relationship between size and function provides little to no information about the *slope* of the relationship between cell size and function within species (Figure 5). Instead, among‐species relationships provide an estimate of the covariance between cell size and *the intercept* (level) of the intraspecific relationship. Thus, it is entirely possible that a positive interspecific size‐function relationship arises from a series of negative intraspecific size‐function relationships (Figure 5). Rather than being synonymous with intraspecific size‐function relationships, interspecific relationships only provide information on the outcome of coevolution between size and function. In other words, intraspecific size‐function relationships describe a current biological process; interspecific relationships describe the evolutionary product of that process that has been shaped over time. Unsurprisingly therefore, the few estimates of intraspecific relationships between unicellular size and function depart both qualitatively and quantitively from those observed among species (Malerba and Marshall 2019; Marshall et al. 2022).

The field's longstanding focus and reliance on interspecific relationships between unicellular size and function means that very few data exist for exploring intraspecific relationships. But we need such data if we are to predict the impacts of warming‐mediated size declines within species. A challenge with robustly estimating intraspecific size‐function relationships in unicells is that the natural size range within any one species is limited. Fortunately, there are several solutions to this problem. For example, it might be valuable to compare strains of the same species that systematically differ in cell size, or to use biogeographical variation in the size of cells of the same species (Marañón et al. 2024). It is important to note, however, that this approach risks confounding other factors—for example, latitudinal variation in temperature could also affect cell function independently of cell size. A more laborious but robust approach is to experimentally manipulate cell size within species, either by artificial selection or experimental evolution (Padfield et al. 2016; Malerba et al. 2018; Marshall et al. 2022) to generate a greater range of cell sizes. Until we have more estimates, our capacity to predict the impacts of warming‐induced changes in size on unicellular function remains constrained.

### 4.1 Beyond Functions—The Relationship Between Cell Size and Demography

Demographic rates (i.e., intrinsic population growth rate, density‐dependence) set the ecological dynamics of all species and their role in communities. As such, an understanding of how size, temperature and demography relate to each other provides a powerful predictive tool with which to forecast the impacts of warming on population dynamics (Hatton et al. 2019; Hillebrand et al. 2022). Metabolic theory does just that: taking the relationships between size, temperature and metabolic rate to predict demographic outcomes.

Classic metabolic theory makes straightforward predictions about the growth rate of microbial populations (Savage et al. 2004): the intrinsic rate of growth (*r*) should increase with temperature and decrease with cell size (*r α* cell size^*β*−1^). The positive relationship between *r* and temperature arises because warmer temperatures (below temperatures that are stressful) tend to increase metabolic rate, allowing more biological work to be done such that cells can divide more frequently (Marshall et al. 2022). The negative effect of cell size on *r* arises because larger cells usually contain more material than smaller cells (but see Marshall et al. 2022). Larger cells require more metabolic work to produce while having lower mass‐specific production rates such that cell divisions are less frequent. Consequently, warmer temperatures increase intrinsic rates of population growth via two mechanisms: increasing metabolic rate and decreasing cell size, which combine to increase *r* with temperature. It is important to note that this theory implicitly assumes that mortality rates are unaffected by size and temperature, so it is only relevant within non‐stressful temperature ranges.

The effects of temperature and cell size on maximum cell density (carrying capacity, or *K*) are more complex, and predictions differ depending on the theory that is used to derive *K*. On the one hand, classic metabolic theory (Savage et al. 2004) predicts that *K* should negatively covary with metabolic rate, and hence with cell size and temperature, according to the equation:

(6) $$K\propto R^{-1}\propto e^{-\delta T}M^{-\beta }$$

where *δ* is the temperature dependency of metabolic rate and *T* is temperature (Bernhardt et al. 2018). This formulation has intuitive appeal—larger or warmer cells have higher metabolic demands such that they will exhaust the available resources more quickly than smaller or colder cells: thus, we expect inverse relationships between *K* and size, and *K* and temperature. Plasticity in cell size complicates these expectations but overall, warming should reduce carrying capacities (see Supporting Information for further details); a prediction that has some empirical support (Bernhardt et al. 2018).

But classic metabolic theory overlooks a crucial element: the effect of *r* on *K*. As has been pointed out (Mallet 2012; Marshall et al. 2023), it is tempting to view *K* as an independent parameter from *r*, but in reality, *K* is a function of *r*:

(7) $$K=\frac{r}{\alpha }$$

where *α* is the intraspecific competition coefficient. Hence, unless α increases superlinearly with *r*, *K* will actually covary positively with *r* (Marshall et al. 2023). Increasing temperatures might therefore increase the strength of competition (i.e., increase *α*), but if warmer temperatures also increase *r* (as is predicted and routinely found), then *r* will act to increase *K*. Thus, unless *α* rises more steeply with temperature than *r*, we would predict that warming has no effect on (or even slightly increases) *K*—a radical departure from classic metabolic theory (see Supporting Information for further details). For example, consider a scenario where warming: (i) decreases cell size; (ii) increases *r*; but (iii) weakly affects *α*. In this scenario, higher temperatures will increase the carrying capacities of unicellular populations, the opposite trend to that predicted by classic metabolic theory. The dependence of *K* on *r* might explain why some empirical studies fail to find the expected negative covariance between *K* and temperature in unicells (Huete‐Stauffer et al. 2015; Arandia‐Gorostidi et al. 2017; Labban et al. 2021). As far as we are aware, very few studies have examined the temperature dependence of *α* (but see DeLong and Lyon 2020): thus the fundamental relationships between temperature, size and demography in unicells remain surprisingly uncertain, limiting our capacity to predict even the direction of the effect of warming on unicellular populations.

## 5 Why Do Cells Change Size—Towards Unicellular Life‐History Theory

If we are to predict the causes and consequences of changes in unicellular size, we require a robust theory base for making these predictions, particularly with regards to projecting future changes to cell size (Czarnoleski and Verberk 2025). Attempts to reveal the drivers of cell size have used a range of modelling approaches that vary in complexity. Some models give primacy to physical constraints, and focus on a single resource (e.g., oxygen [Deutsch et al. 2022]). Such approaches might indicate the outer limits of cell size variation, or the ‘hard’ constraints on cell size, but we suspect that most biological variation exists within these limits. In contrast, models that emphasise evolutionary processes (c.f. constraints) seem more likely to accommodate the diversity of cell sizes that we observe in nature and better predict their future trajectories. Thus, some sort of optimisation of cell size, as it connects to various fitness‐enhancing functions seems necessary. For example, Leles and Levine (2023) assume that the surface of phytoplankton cells limits transportation rates of molecules into the cell; while cellular volume limits intracellular processes, such as photosystems and storage. Because nutrient uptake is less temperature‐dependent than metabolic rate, Leles and Levine (2023) predict that cells must increase surface area to volume ratios as temperatures increase to accommodate more transport, thereby reducing cell size. In other words this model predicts the observed negative covariance between cell size and temperature (Leles and Levine 2023). Nevertheless, even this optimality approach implicitly assumes that the trade‐offs that set these optima are themselves fixed, when in reality they can also evolve. For example, the evolution of larger cells can select for a higher density of surface transporters on those cells, negating the loss of relative surface area and maintaining rates of transport that scale linearly with volume (Malerba et al. 2021). Complicating matters further still, cell membranes themselves may account for the majority of metabolic demands such that increasing relative surface area may also increase relative metabolic demands (Czarnoleski and Verberk 2025).

It is also unclear whether optimisation approaches that maximise population growth rates use the appropriate evolutionary currency. For example, in undisturbed systems, evolution may maximise population carrying capacity rather than growth rate (Lande 2009). Likewise, optimisation approaches usually preclude frequency‐dependent selection, when instead it seems likely that the fitness returns of one cell size might depend on the relative size of neighbouring cells. In recognition of this possibility, Adaptive Dynamics Models explore the possibility for evolutionarily stable cell sizes while explicitly modelling frequency‐dependence (Chen et al. 2020). Studies that have employed such modelling approaches for unicells also predict that cell sizes should be smaller under warmer temperatures (Chen et al. 2020). Adaptive Dynamics Models are typically mathematically more complex than optimisation approaches and so necessarily reduce physiological complexity in their underlying fitness functions.

Hence no single modelling approach is perfect (Czarnoleski and Verberk 2025): different approaches emphasise different processes (i.e., mechanistic limits, optimisation or evolutionary games), but all of these processes will likely contribute to cell size variation to some degree. We therefore do not advocate for any particular class of model moving forward, but note that each choice has its (dis)advantages, and none will be perfectly complete (Czarnoleski and Verberk 2025). One guiding principle however should be that increasing model complexity increases the risk of spurious congruence: models that are rich in poorly constrained parameters should be avoided. Thus we would argue that an essential first step is to resolve some of the fundamental cell size‐function relationships, such that we can realistically parameterise predictive theory. For example, recent trait‐based approaches (Wickman et al. 2024) show tremendous promise but as we have shown here, our understanding of many of these trait‐function relationships remains surprisingly incomplete.

## 6 Future Research Priorities

We find preliminary evidence for warmer temperatures reducing cell size within species of bacteria and yeast, but we were surprised by how few studies were available, particularly relative to similar work in protists (Atkinson et al. 2003). Thus, we call for more studies that manipulate temperature and monitor any subsequent changes in cell size in bacteria and yeast (both within and among species), particularly studies that explore both stressful and non‐stressful temperature ranges as theory predicts different size responses across these ranges (Leles and Levine 2023).

Most current estimates of metabolic rate come from relatively artificial conditions: metabolic rates are often measured on samples of cells that are denser than those observed in nature and under unrealistic flow regimes. Estimates of maximum natural cell densities vary between 10^6^ and 10^11^ cells/mL depending on the system (Sherr et al. 2006; Flemming et al. 2016), while the studies in our dataset measured metabolism at densities that ranged from 10^2^ to 10^12^ cells/mL. Similarly, for aquatic unicells, natural water movements will reduce cell boundary layers and facilitate respiration, but the influence of such fluid dynamics is probably minimised in respirometry chambers. While we cannot envision a mechanism by which these artificialities will affect estimates of scaling, they will almost certainly affect metabolic levels (intercepts) such that we may be systematically underestimating metabolic rates of unicells relative to those that occur in the field. Gaining estimates under more realistic density‐, nutrient‐ and hydrodynamic‐regimes will improve our understanding of energy fluxes in unicells.

In light of our finding that ignoring density effects artifactually overestimates metabolic scaling, estimates of other key size‐function relationships, such as photosynthesis and resource uptake, should also be revisited. To explore these effects rigorously, future studies should seek to manipulate the density of cells within species when measuring functional relationships to explore how density influences estimates of scaling both among and within species. Estimating these relationships will resolve key uncertainties with regard to how warming‐induced declines in cell size will affect biological functions.

Finally, and perhaps most urgently, we need more estimates of how variation in unicellular size affects function specifically within species. Despite evidence from every clade and habitat that cells are changing size within species, only a handful of studies have explored intraspecific relationships between size and function. Unfortunately, we cannot rely on among‐species patterns to predict within‐species size‐function relationships. While generating sufficient variation in size within species is a nontrivial challenge, we believe such approaches are crucial if we are to accurately predict the consequences of size declines in unicells.

## Author Contributions

D.J.M. collected the data and did the analyses; C.R.W. did the phylogenetic analyses and figure preparation. D.J.M. wrote the first draft and all authors contributed substantially to revisions.

## Supporting information


**Figure S1:** A replotting of Figure 1 to show species‐level (left panel) and strain‐level (right panel) effects of temperature on cell size.
**Figure S2:** Relationship between cell size and the cell sample density used in respirometry studies of metabolic rate in unicellular organisms.
**Table S1:** Studies included in the preliminary analysis of the effects of temperature on cell size in bacteria and yeast.
**Table S2:** Summary table of major compilations of metabolic scaling data for unicellular species measured in the laboratory.

**Figure S3:** Imputed sample densities based on the size‐specific relationships that we observed for those species for which these data were available.

## Data Availability

All data used in the main analyses of metabolic scaling are available at: https://doi.org/10.5281/zenodo.18333504. The data for the size changes in bacteria are included as Supporting Information. The data were analysed using Systat Vers 13.2 so no code can be made available but the analyses are fully described and are very simple (multiple regressions).
