# Supplementary figures and images for: Drivers and Consequences of Size Declines in Unicells
Source: Ecol Lett. 2026 May 5;29:e70387. doi: 10.1111/ele.70387 (PMC13146120; doi:10.1111/ele.70387)

Figure S1


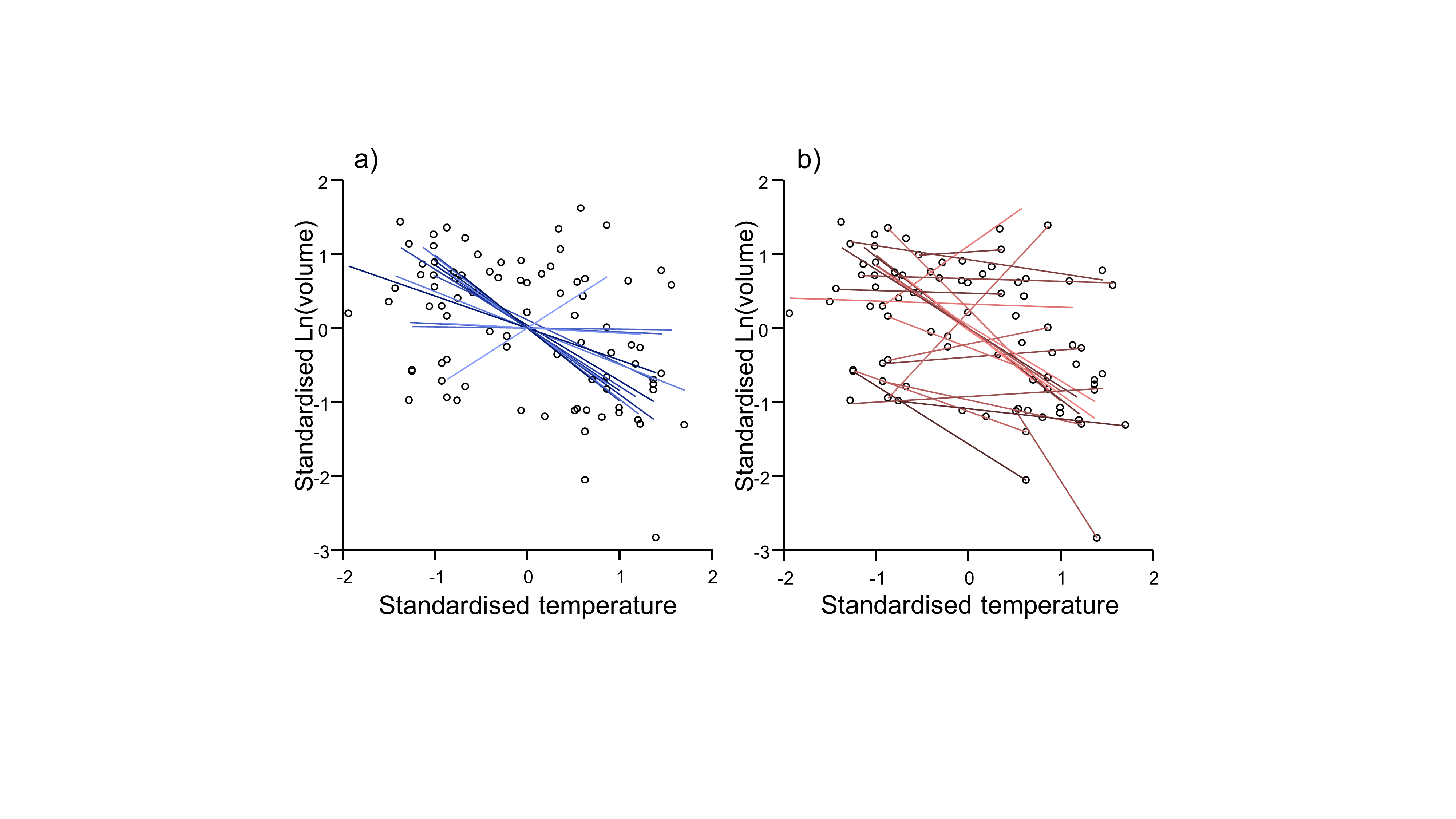


Figure S2


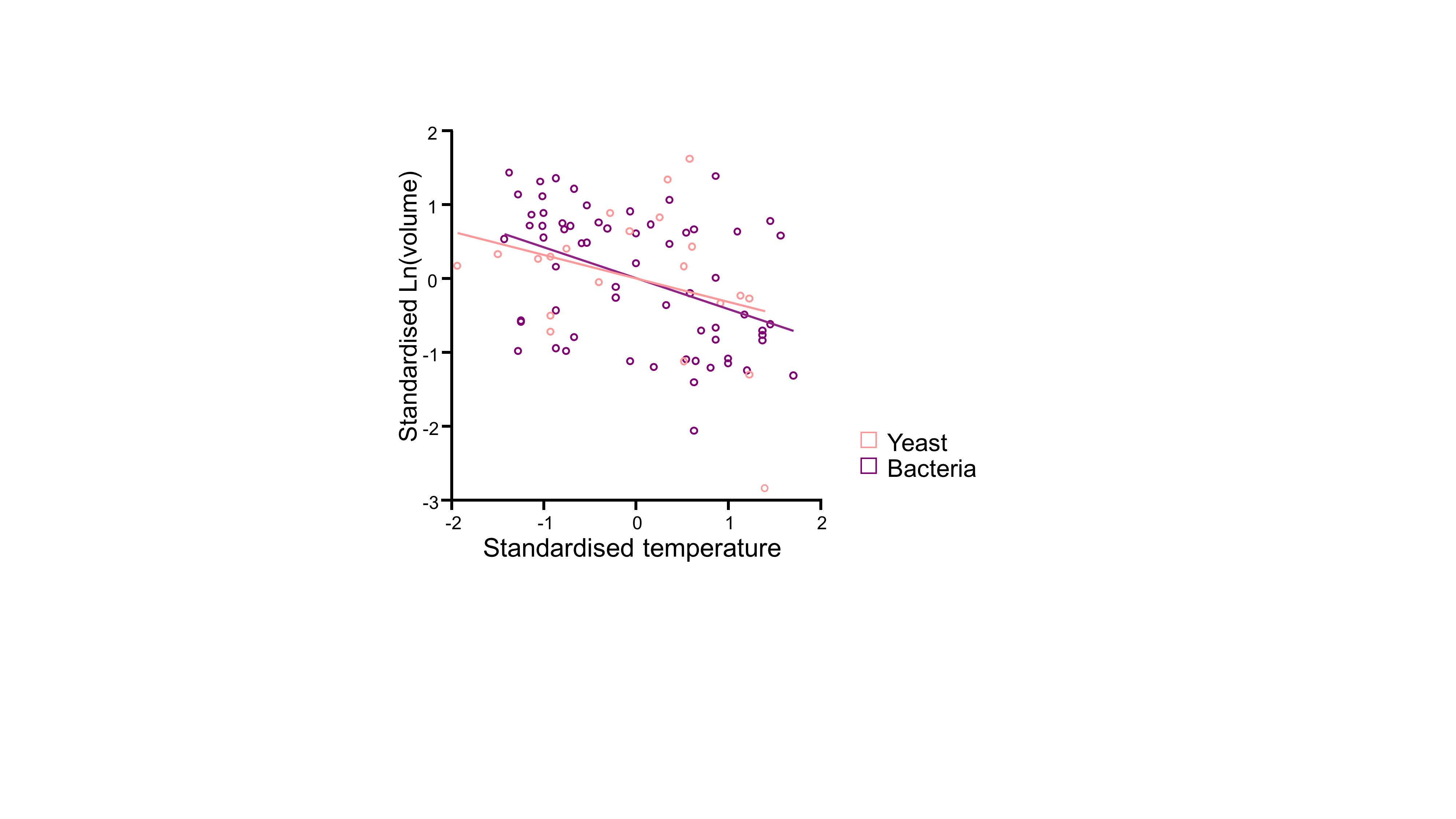


Figure S3


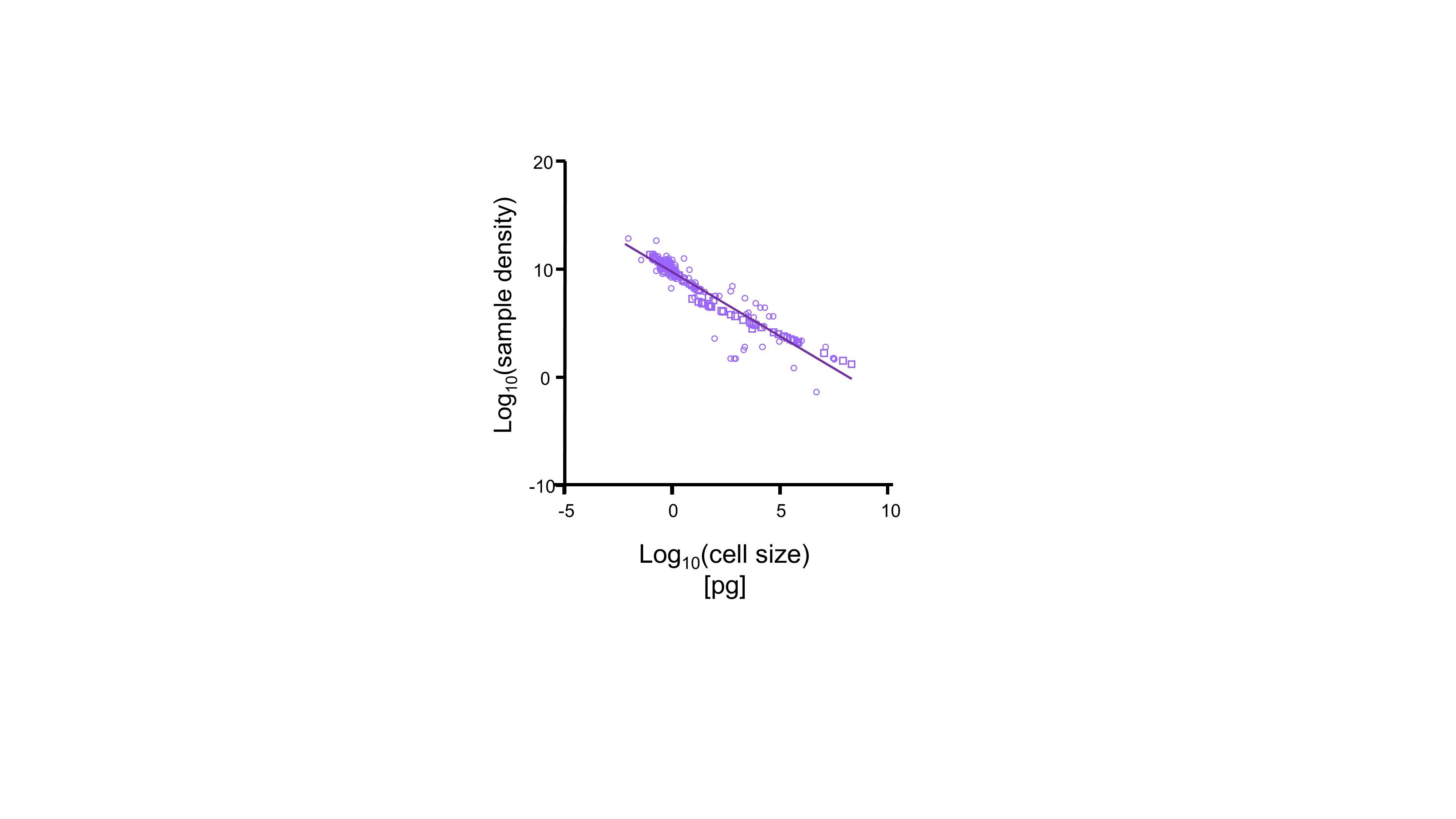

Supplement: Supplementary file 1 — Figure S1: A replotting of Figure 1 to show species‐level (left panel) and strain‐level (right panel) effects of temperature on cell size. Figure S2: Relationship between cell size and the cell sample density used in respirometry studies of metabolic rate in unicellular organisms. Table S1: Studies included in the preliminary analysis of the effects of temperature on cell size in bacteria and yeast. Table S2: Summary table of major compilations of metabolic scaling data for unicellular species measured in the laboratory. [file ELE-29-0-s001.docx]

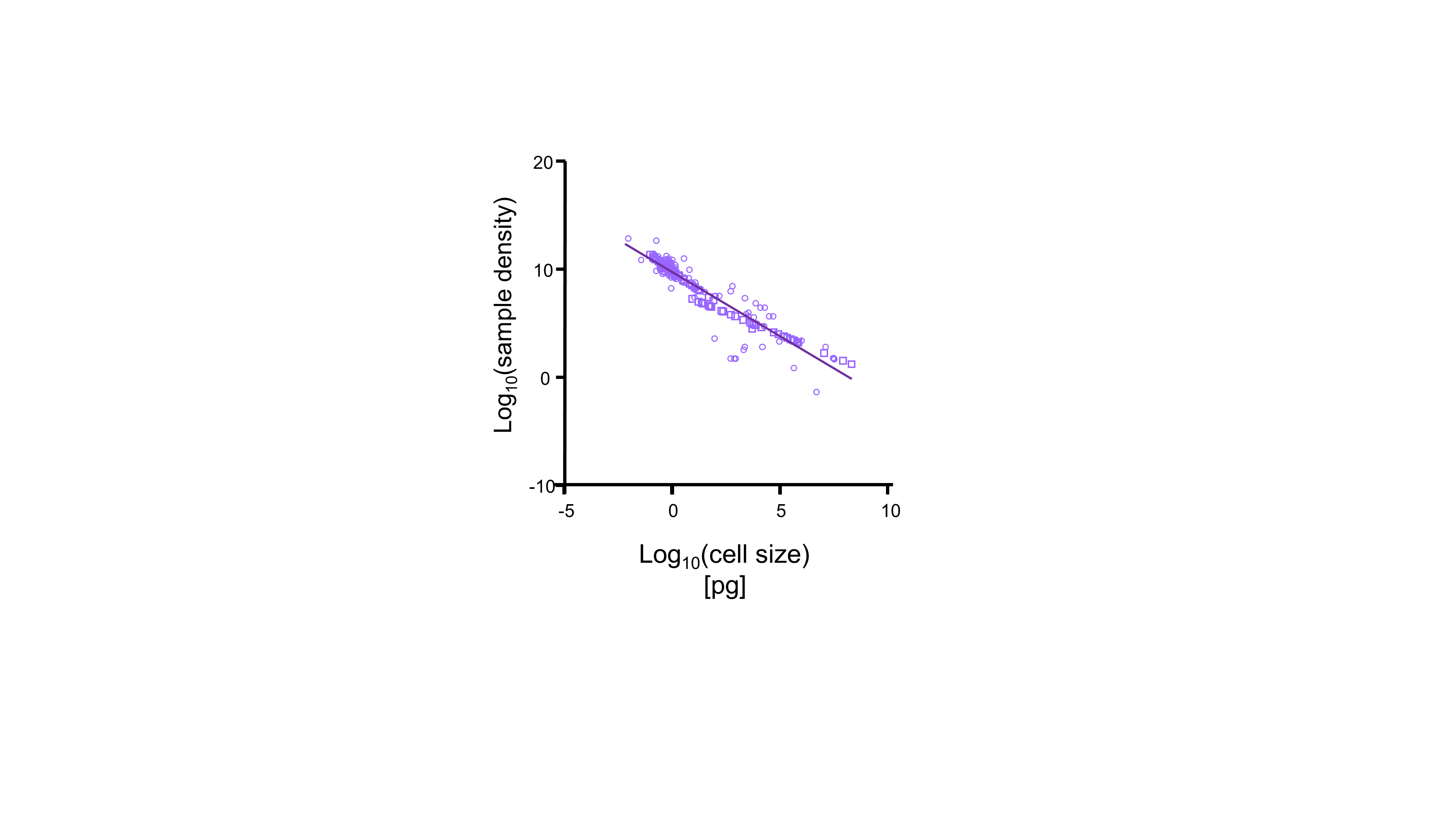

Supplement: Supplementary file 2 — Figure S3: Imputed sample densities based on the size‐specific relationships that we observed for those species for which these data were available. [file ELE-29-0-s002.tif]
